# Death Following Ingestion of an Edible Marijuana Product — Colorado, March 2014

**DOI:** 10.15585/mmwr.mm6428a6

**Published:** 2015-07-24

**Authors:** Jessica B. Hancock-Allen, Lisa Barker, Michael VanDyke, Dawn B. Holmes

**Affiliations:** 1 Epidemic Intelligence Service, CDC; 2 Colorado Department of Public Health and Environment; 3 Denver Office of the Medical Examiner

In March 2014, the Colorado Department of Public Health and Environment (CDPHE) learned of the death of a man aged 19 years after consuming an edible marijuana product. CDPHE reviewed autopsy and police reports to assess factors associated with his death and to guide prevention efforts. The decedent’s friend, aged 23 years, had purchased marijuana cookies and provided one to the decedent. A police report indicated that initially the decedent ate only a single piece of his cookie, as directed by the sales clerk. Approximately 30–60 minutes later, not feeling any effects, he consumed the remainder of the cookie. During the next 2 hours, he reportedly exhibited erratic speech and hostile behaviors. Approximately 3.5 hours after initial ingestion, and 2.5 hours after consuming the remainder of the cookie, he jumped off a fourth floor balcony and died from trauma. The autopsy, performed 29 hours after time of death, found marijuana intoxication as a chief contributing factor. Quantitative toxicologic analyses for drugs of abuse, synthetic cannabinoid, and cathinones (“bath salts”) were performed on chest cavity blood by gas chromatography and mass spectrometry. The only confirmed findings were cannabinoids (7.2 ng/mL delta-9 tetrahydrocannabinol [THC] and 49 ng/mL delta-9 carboxy-THC, an inactive marijuana metabolite). The legal whole blood limit of delta-9 THC for driving a vehicle in Colorado is 5.0 ng/mL. This was the first reported death in Colorado linked to marijuana consumption without evidence of polysubstance use since the state approved recreational use of marijuana in 2012.

According to the police report, the decedent had been marijuana-naïve, with no known history of alcohol abuse, illicit drug use, or mental illness. In addition to listing inactive ingredients, the cookie label described the psychoactive ingredients as “65 mg THC/6.5 servings (THC, tetrahydrocannabinol, the principal psychoactive agent in cannabis).” The label also noted, “This marijuana product has not been tested for contaminants or potency.” According to the police report, the sales clerk had instructed the buyer and decedent to divide each cookie into sixths, each piece containing approximately 10 mg of THC, the serving size, and to ingest one serving at a time. The police report did not indicate whether the sales clerk provided specific instructions for how long to wait between ingesting each serving.

This case illustrates a potential danger associated with recreational edible marijuana use. Some studies have suggested an association between cannabis and psychological disturbances (1). Second to alcohol, marijuana is the most commonly used recreational drug in the United States, with an estimated 19.8 million past-month users during 2013 (2). In 2012, Colorado and Washington became the first states to permit recreational use of marijuana under their state laws (3). The first state-licensed recreational marijuana stores in Colorado opened in January 2014. An estimated 45% of Colorado’s marijuana sales involve edible marijuana, including THC-infused food, drink, and pills (4,5). Colorado’s marijuana surveillance system collects adverse outcomes data from hospitalizations, emergency department visits, and poison center calls.

Systemic THC levels and psychoactive effects after ingestion are highly variable because of differences in bioavailability, rate of gastrointestinal absorption, and metabolic first-pass effect whereby an orally administered drug is partially metabolized (principally in the liver) before reaching systemic distribution (6,7). Because absorption is slower, the onset of effects is delayed (with mean peak plasma concentration at 1–2 hours after ingestion, in contrast with 5–10 minutes to peak plasma concentrations if smoked), and duration of intoxication is longer when THC is ingested compared with when it is smoked (7). Whereas a single-serving recreational edible marijuana dose in Colorado was set at 10 mg of THC, multiple-dose recreational edible products, often containing 100 mg of THC, were available during March 2014 (4). The marijuana store where the implicated cookies had been purchased voluntarily gave all 67 remaining cookies of the same brand to the Denver Police Department. Testing confirmed that the THC levels in the items were within required limits. Because of the delayed effects of THC-infused edibles, multiple servings might be consumed in close succession before experiencing the “high” from the initial serving, as reportedly occurred in this case. Consuming a large dose of THC can result in a higher THC concentration, greater intoxication, and an increased risk for adverse psychological effects.

Recreational marijuana is now permitted for persons aged ≥21 years under state law in four states (Alaska, Colorado, Oregon, and Washington) and the District of Columbia; marijuana-attributed morbidity and mortality surveillance can help guide efforts to prevent overconsumption in these jurisdictions. Regulation of recreational marijuana edibles in Colorado continues to evolve. On the basis of initial surveillance data in Colorado and numerous cases of accidental overconsumption, on February 1, 2015, Colorado instituted new packaging and labeling rules, requiring that recreational edible marijuana products contain no more than 10 mg of THC, or have clear demarcation of each 10-mg serving (8). In addition, before distribution, cannabinoid potency testing is now performed on batches of recreational edible marijuana products by state-certified laboratories. Other states permitting recreational marijuana use could potentially reduce adverse health effects by considering similar THC limits in marijuana edible products, and by enforcing clear labeling standards that require information on multidose products. Although the decedent in this case was advised against eating multiple servings at one time, he reportedly consumed all five of the remaining servings of the THC-infused cookie within 30–60 minutes after the first serving, suggesting a need for improved public health messaging to reduce the risk for overconsumption of THC.
